# Magnetic control of graphitic microparticles in aqueous solutions

**DOI:** 10.1073/pnas.1817989116

**Published:** 2019-01-25

**Authors:** Johnny Nguyen, Dario Valter Conca, Johannes Stein, Laura Bovo, Chris A. Howard, Isabel Llorente Garcia

**Affiliations:** ^a^Department of Physics and Astronomy, University College London, London WC1E 6BT, United Kingdom;; ^b^London Centre for Nanotechnology, University College London, London WC1H 0AJ, United Kingdom;; ^c^Department of Innovation and Enterprise, University College London, London W1T 4TJ, United Kingdom

**Keywords:** label-free magnetic transport, submerged micrographite, diamagnetic manipulation, magnetophoresis, HOPG

## Abstract

Graphite is an inexpensive material with useful electrical, magnetic, thermal, and optical properties. It is also biocompatible and used universally as a substrate. Micrometer-sized graphitic particles in solution are therefore ideal candidates for novel lab-on-a-chip and remote manipulation applications in biomedicine, biophysics, chemistry, and condensed-matter physics. However, submerged graphite is not known to be amenable to magnetic manipulation, the optimal manipulation method for such applications. Here, we exploit the diamagnetism of graphite and demonstrate contactless magnetic positioning control of graphitic microflakes in diamagnetic aqueous solutions. We develop a theoretical model for magnetic manipulation of graphite microflakes and demonstrate experimentally magnetic transport of such particles over distances ∼200 μm with peak velocities ∼15 μm/s in inhomogeneous magnetic fields. We achieve fully biocompatible transport for lipid-coated graphite in NaCl aqueous solution, paving the way for previously undiscovered biomedical applications. Our results prove that micrometer-sized graphite can be magnetically manipulated in liquid media.

## Significance

This paper presents the magnetic transport of diamagnetic graphite microparticles in water solutions. Given the dominance of viscous drag forces at the microscale, moving a microparticle that is submerged in liquid is comparably as hard as moving a macroparticle within dense honey. While diamagnetism is a weak magnetic property, for graphite it can be exploited to generate useful transport in liquid. The contactless magnetic control of biocompatible micrographite, together with graphite’s unique physical properties, opens up possibilities for applications in sensing, analysis, synthesis, and diagnosis in chemistry, biology, medicine, and physics.

The controlled transport of microparticles in solution is key to a broad range of microfluidic and lab-on-a-chip applications (1, 2). These include microreactors for chemical analysis and synthesis (3–5); biosensing platforms for drug discovery and clinical diagnosis (6–9); and separation and sorting of cells, bacteria, and/or viruses in mixtures for therapy and diagnosis (e.g., refs. 10–14). In some of these applications microparticles can be used as reaction substrates or as specific labels for analytes, with particle transport introducing the ability of, e.g., controlled sample preparation, actuation from a reaction area to a detection region, and isolation for assay enhancement (15, 16). For micrometer-sized particles in a static liquid, the generation of particle motion requires that large, dominant viscous drag forces are overcome (low Reynolds number regime). Magnetic transport is the preferred particle transport technique for biomedical/chemical applications as it is insensitive to the solution’s conductivity and ionic composition, does not induce electrochemistry, and does not perturb biological function (17). The magnetic control of submerged microparticles is also highly relevant to the field of small-scale robotics (17, 18).

Magnetic manipulation and actuation schemes for microparticles have been mostly limited to ferromagnetic, paramagnetic, and superparamagnetic particles (17, 19). However, these suffer several limitations: Attraction toward the magnetic-field source can preclude fully controlled contactless transport, particles cannot be stably trapped in 3D solely with static magnetic fields, and quantification of the magnetic moment for quantitative sensing applications can be challenging.

On the other hand, diamagnetic microparticles (attracted to magnetic-field minima) can be made to follow tailor-designed minimum-field paths at a distance from the magnetic source and can be confined in 3D with time-independent magnetic fields. Another advantage is the fact that the induced magnetic moment is approximately independent of particle shape for all practical purposes, greatly simplifying the quantification of magnetic potential energy, force, and torque. The manipulation in solution of purely diamagnetic microobjects can be extremely powerful and versatile; however, to date, it has been explored only by a few research groups. Experiments have often required fluid flow to assist trapping or manipulation and studies have been limited to certain biological cells in culture media and to cells and polystyrene beads in paramagnetic salt solutions, in ferrofluids (not always fully biocompatible), or in dispersions of superparamagnetic nanoparticles (e.g., refs. 14 and 19–25). It is also possible to achieve magnetic transport of diamagnetic microobjects via magnetic labeling with para/ferromagnetic nanoparticles (e.g., refs. 12, 26, and 27). Here, we focus on label-free magnetic transport of diamagnetic graphite microflakes in absence of fluid flow.

Graphite, and specifically highly oriented pyrolytic graphite (HOPG), is one of the most strongly diamagnetic materials known (28, 29), a quality that remains largely unexploited. HOPG is reasonably low cost and widely used as an ideal substrate. HOPG microparticles can be easily produced by low-power sonication and can be lipid coated for dispersion in aqueous solution (30). HOPG also has useful electrical properties (strong conductivity and polarizability) and interesting optical and thermal properties (e.g., broadband absorption and bolometric photoresponse—heating and resistance change—upon infrared absorption) (31–36). HOPG is magnetically and electrically anisotropic and, hence, microflakes can be oriented and rotationally trapped in solution, as recently demonstrated (30, 37). The physical properties of HOPG make it ideal for rich microparticle manipulation schemes such as combinations of transport, trapping, and orientation and/or combinations of magnetic, electrical, and optical manipulation and/or detection (e.g., refs. 11 and 30).

Furthermore, HOPG surfaces, similarly to graphene, can be chemically modified in various ways to improve particle solubility and function: e.g., graphene has been functionalized with polymers (e.g., PEG), self-assembled peptides, proteins (e.g., antibodies), peptide nucleic acids, and DNA (38–46). HOPG is biocompatible and HOPG surfaces have been tested as mammalian cell substrates (47). Various studies have shown that graphene substrates are suitable for the growth and proliferation of various mammalian cells, including stem cells, and that such substrates can promote cell adhesion and proliferation, gene transcription and expression, and stem cell differentiation (e.g., refs. 38 and 48–51). As HOPG surfaces have comparable structure and chemical reactivity to graphene, these observations likely also apply to HOPG substrates. Hence, HOPG microflakes have broad potential for the development of biomedical/chemical applications in solution involving DNA, protein markers, living cells, and bacteria, etc.

In this paper, we report the contactless magnetic transport of graphitic (HOPG) microparticles in static aqueous solutions as a step toward full 3D magnetic positioning control. This is one of very few reports of magnetic transport of diamagnetic microparticles in diamagnetic solutions (as opposed to ferro/paramagnetic solutions). Using NdFeB permanent magnets to generate a spatially inhomogeneous magnetic field, we demonstrate unidirectional magnetophoresis of individual HOPG microflakes. We develop a theoretical model for the magnetophoresis of submerged anisotropic diamagnetic microparticles and present magnetic transport velocity data for HOPG microflakes dispersed in two types of diamagnetic solutions: uncoated HOPG in an acetone–water mixture and lipid-coated HOPG in an aqueous NaCl solution. The latter demonstrates fully biocompatible diamagnetic transport (as opposed to some schemes in paramagnetic/ferrofluid solutions). We achieve HOPG magnetophoretic velocities up to ∼15 μm/s over transport distances ∼200 μm. These velocities are ∼10-fold higher compared with those previously reported for diamagnetic cells in diamagnetic solutions (14). Our results prove that graphite microparticles can be magnetically manipulated in solution. This work can open unexplored avenues for improved manipulation schemes exploiting the full potential of graphite and diamagnetic control and reducing the need for flow control in the abovementioned applications.

## Results

### Graphitic Microflakes in Solution.

HOPG is a highly ordered, synthetic carbon polycrystal. It is made of many crystallites, each of which is a stack of parallel graphene planes with an out-of-plane direction termed the *c* axis. The *c* axes of the different crytallites are parallel to each other within a few degrees or less, making a highly oriented material with a low mosaic spread (low *c*-axis dispersion). In this work, we use HOPG with a mosaic spread of 0.4°±0.1° and typical crystallite sizes of a few micrometers in plane and exceeding 1 μm out of plane (52, 53).

HOPG is strongly diamagnetic and magnetically anisotropic. We measure a large negative magnetic susceptibility along its out-of-plane direction, perpendicular to the graphene planes [$\chi_{⊥}=\left(-5.82\pm 0.01\right)\times 10^{-4}$], and a lower one along its in-plane direction [$\chi_{∥}=\left(-8.2\pm 0.1\right)\times 10^{-5}$]. These values are measured with a superconducting quantum interference device (SQUID) at 295 K for the 1-mm-thick HOPG bulk used to prepare microflakes (*Materials and Methods* and *SI Appendix*, Note 1).

We prepare micrometer-sized HOPG flakes by means of low-power bath sonication (*Materials and Methods*). We use HOPG microflakes with full sizes ∼4.6 μm×3.4 μm×1.2 μm on average. Dimensions for all particles used in experiments can be found in *SI Appendix*, Note 2.

Compared with chemical or electrochemical exfoliation, it has been shown that bath sonication is better at preserving the carbon-stacking structure of HOPG (54). To evaluate possible structural modifications in the sonicated microflakes, we acquire Raman spectra for the HOPG bulk and for individual representative HOPG microflakes. A moderate increase in disorder is observed for the sonicated microflakes compared with the bulk. This is consistent with a transition to nanocrystalline graphite (smaller crystallite domains compared with pristine HOPG) for the microflakes and with increased surface roughness after sonication, with the possible additional presence of point defects and/or edge defects but with minimal damage to the graphite lattice (*Materials and Methods* and *SI Appendix*, Note 3) (55–58).

HOPG microparticles are strongly hydrophobic and aggregate in aqueous solution. To prevent this aggregation and produce dilute HOPG dispersions, we take two approaches: (*i*) Uncoated HOPG microflakes are naturally dispersed in a mixture of acetone and water (40−60% volume fractions, respectively) (59), or (*ii*) HOPG microflakes are coated with a thin POPC lipid shell and dispersed in 20 mM NaCl aqueous solution (*Materials and Methods*) (30).

### Theoretical Model of Magnetic Manipulation of Anisotropic Diamagnetic Microparticles.

In this section, we develop a theoretical model for the magnetic manipulation in solution of diamagnetic microparticles that are magnetically anisotropic, focusing on magnetophoretic transport of submerged HOPG microflakes. The theoretical framework for magnetically isotropic microspheres has been provided elsewhere (19, 60).

In the presence of an applied static magnetic field $B_{0}$, a submerged diamagnetic particle acquires an effective induced magnetic moment given by

$$m_{e\mathrm{ff}}\approx \frac{V}{\mu_{0}}\left(\chi_{2}-\chi_{1}\right)\cdot B_{0}\;,$$ [1]

where V is the particle volume, $\mu_{0}$ is the permeability of free space, and $\chi_{2}$ and $\chi_{1}$ are the volume magnetic susceptibility tensors for particle and fluid, respectively. The approximation in Eq. **1** is valid only for weakly magnetic particles with |χ|≪1 and hence specifically for diamagnetic particles (χ<0, typically $|\chi |<10^{-4}$). It is based on the fact that the magnetic field inside the particle can be considered approximately equal to the external magnetic field. For particles with |χ|≳1 (e.g., ferromagnetic particles and some superparamagnetic particles), this is not the case, as particle magnetization is significant and geometrical demagnetizing factors need to be considered (details in *SI Appendix*, Note 4). A key consequence of this approximation is that particle shape and geometry have a negligible effect on the effective magnetic moment induced on the particle. Hence, diamagnetic manipulation presents the advantage of enabling straightforward quantification.

**Fig. 1.**
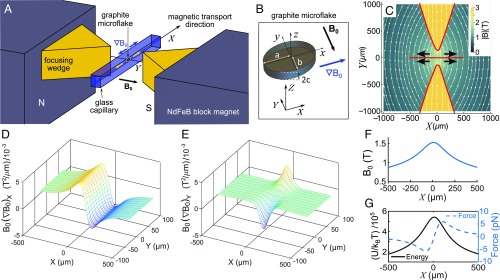
Magnetic transport of graphite microflakes in solution. (*A*) Schematic of experimental setup with NdFeB permanent magnets, focusing steel wedges and glass capillary with submerged HOPG microflake (not to scale). Microflake motion is mainly along X (yellow arrows), following the main field gradient. (*B*) Schematic HOPG microflake as an elliptical disk with half-sizes a, b, and c. Axes (x,y,z) and (X,Y,Z) define particle and laboratory frames of reference, respectively. (*C*) Top view and map of calculated magnetic-field strength and field lines in the gap between the focusing wedges. Black arrows indicate the direction of transport for diamagnetic HOPG microparticles. (*D* and *E*) Corresponding maps of calculated $B_{0}{\left(\nabla B_{0}\right)}_{X}$ and $B_{0}{\left(\nabla B_{0}\right)}_{Y}$ on the XY plane. (*F*) Calculated magnetic-field strength along X (at Y=0). (*G*) Calculated magnetic potential energy (left axis, solid line, in units of thermal energy $k_{B}T$) and magnetic force (right axis, dashed line) along X exerted on a representative HOPG microflake with half-sizes ∼3μm, ∼2μm, and ∼1μm.

The magnetic susceptibility tensors can be expressed in the particle frame of reference (Fig. 1*B*) as

$$\chi_{1}=\left(\begin{array}{ccc} \chi_{1} & 0 & 0\\ 0 & \chi_{1} & 0\\ 0 & 0 & \chi_{1} \end{array}\right)\;,\;\chi_{2}=\left(\begin{array}{ccc} \chi_{2,∥} & 0 & 0\\ 0 & \chi_{2,∥} & 0\\ 0 & 0 & \chi_{2,⊥} \end{array}\right)\;,$$ [2]

where $\chi_{1}$ is the isotropic magnetic susceptibility of the fluid, and $\chi_{2,∥}$ and $\chi_{2,⊥}$ are the in-plane and out-of-plane components of the anisotropic magnetic susceptibility of the HOPG particle, respectively. The direction of the applied magnetic-field vector $B_{0}$ with respect to the particle’s principal axes is given by angles θ and ϕ so that $B_{0}$ can be expressed as $B_{0}=B_{0}\left(\sin\theta \cos\phi \;\hat{x}+\sin\theta \sin\phi \;\hat{y}+\cos\theta \;\hat{z}\right)$, where $B_{0}$ is the field amplitude and the unitary vectors $\hat{x}$, $\hat{y}$, and $\hat{z}$ correspond to the particle frame of reference (*SI Appendix*, Note 4). The magnetic potential energy can be calculated as

$$\begin{array}{ccc} U_{m} & = & -\overset{B_{0}}{\underset{0}{\int }}m_{e\mathrm{ff}}\left(B\right)\cdot dB\\ & = & -\frac{VB_{0}^{2}}{2\mu_{0}}\left[\left(\chi_{2,∥}-\chi_{1}\right)+\left(\chi_{2,⊥}-\chi_{2,∥}\right)\cos^{2}\theta \right]\;, \end{array}$$ [3]

and the magnetic force derived from the potential is $F_{m}=-\nabla U_{m}$, which depends on the particle orientation (θ) with respect to the field (*SI Appendix*, Note 4). For magnetically anisotropic HOPG microflakes in aqueous solution it is energetically favorable to orient with the graphene planes parallel to the magnetic-field lines (θ=±90°), as demonstrated in the presence of field amplitudes ∼200 mT (30). For particles oriented in this way, the magnetic force becomes

$$F_{m}=\frac{V}{\mu_{0}}\left(\chi_{2,∥}-\chi_{1}\right)B_{0}\nabla B_{0}\;.$$ [4]

This magnetic force moves the microparticles in solution and is opposed by the viscous drag force, $F_{visc}$. The latter has components $F_{visc,k}=-\gamma_{k}v_{k}$, where $\gamma_{k}$ is the viscous drag coefficient along direction k for the particles and $v_{k}$ is the particle velocity along direction k (k=x, y, z). As a convenient general approximation, we model HOPG microflakes as elliptical disks (Fig. 1*B*) as this enables the use of analytical expressions for the viscous drag coefficients (*Materials and Methods*). In the equation of motion, inertial forces are negligible compared with viscous forces (low Reynolds number regime) and the effect of thermal Brownian fluctuations can be neglected as particles undergo directed motion (*Materials and Methods*). For horizontal motion (k=x,y: no gravitational or buoyant forces) and in absence of fluid flow, the equation of motion is hence given by the balance of magnetic and viscous drag forces, $F_{m}+F_{visc}=0$, and the magnetophoretic velocity components can be obtained as

$$v_{k}=\frac{V}{\mu_{0}\gamma_{k}}\left(\chi_{2,∥}-\chi_{1}\right)B_{0}{\left(\nabla B_{0}\right)}_{k}\;.$$ [5]

Graphitic flakes can be magnetically transported by designing magnetic landscapes with large enough field amplitude and field gradient ($B_{0}\nabla B_{0}$) along a desired path. In general, the direction of motion of the particle in the fluid depends on the sign of the difference in magnetic susceptibility between particle and fluid ($\chi_{2,∥}-\chi_{1}$). For a particle more diamagnetic than its environment [$\left(\chi_{2,∥}-\chi_{1}\right)<0$], the particle moves in the direction opposite to the field gradient, i.e., toward magnetic-field minima, to minimize its magnetic interaction energy (Eq. **3**). This is the case for HOPG microflakes in water as well as for diamagnetic particles in paramagnetic solutions (e.g., polystyrene beads in ${MnCl}_{2}$ aqueous solution). On the other hand, for a particle more paramagnetic than its environment [$\left(\chi_{2}-\chi_{1}\right)>0$] (e.g., paramagnetic particle in diamagnetic solution), motion is toward magnetic-field maxima, i.e., typically toward the magnetic source.

### Experimental Setup, Data Acquisition, and Image Analysis.

We generate a strong magnetic field and field gradient to magnetically transport HOPG microflakes in solution, using two NdFeB permanent magnet blocks (grade N50M NdFeB, 25×25×20 mm, part no. NIBL01484; MagnetSales) (magnet characterization in *SI Appendix*, Note 5). The magnets are placed with opposite poles facing each other at a distance of 12 mm and two custom-made wedge-shaped steel pieces (4 mm×3 mm base, 6 mm height) are used to concentrate the magnetic-field lines, with a gap between their tips of ∼270 μm along Y (Fig. 1 *A* and *C*). A thin glass capillary (squared cross-section, 100 μm inner width, 50-μm-thick walls) holds the solution with microparticles, stretching along the horizontal X direction in the gap between the wedges (Fig. 1*A*). The wedge-shaped steel focusing pieces ensure an approximately uniform magnetic field and field gradient across the vertical dimension of the capillary. A photograph of the setup can be found in *SI Appendix*, Note 6.

The magnetic-field lines resulting from the magnet–wedge arrangement are shown in Fig. 1*C*, modeled using the Radia package for Mathematica (*Materials and Methods*) (61). The magnetic-field strength is maximum in the central gap between the wedge tips ($B_{0}∼1.5\;T$ at X=0, Fig. 1*F*) and a large field gradient up to 2,300 T/m is generated along X. HOPG microparticles move away from the central position (X=0) along X as the field decays away in the direction indicated by the black arrows in Fig. 1*C*. The magnetophoretic velocity (Eq. **5**) for a particle in its transport path changes mainly according to the change in $B_{0}{\left(\nabla B_{0}\right)}_{k}$. Calculated maps of $B_{0}{\left(\nabla B_{0}\right)}_{X}$ and $B_{0}{\left(\nabla B_{0}\right)}_{Y}$ vs. X and Y are shown in Fig. 1 *D* and *E*. It is evident from these maps that the main magnetophoretic motion is along X and that the chosen magnetic configuration also generates a focusing effect toward the capillary axis near X=0 as a small field gradient along Y acts on particles located off axis. Fig. 1*G* shows the calculated magnetic force along X in the piconewton level (Eq. **4**) and magnetic potential energy (antitrapping) (Eq. **3**) acting in theory on a HOPG microflake submerged in water–acetone solution (*Materials and Methods*). Magnetic forces along the vertical Z direction are negligible in this setup (there is no magnetic manipulation along Z).

In the experiments, each single particle is transported multiple times to either side of X=0. Different transport tracks are obtained by moving the capillary to place the particle again between the wedge tips (X=0) before measuring each time. A custom-built microscope with a 10×-magnification objective [Olympus PLN 10×, 0.25 numerical aperture (NA), 10.6 mm working distance] (*SI Appendix*, Note 6) and a CMOS camera records movies (at 13 frames per second) of individual microparticles as they move. Image processing via single-particle–tracking algorithms custom written in Matlab (*Materials and Methods*) is used to extract the particle’s dimensions (*SI Appendix*, Note 2), its center-of-mass position on the XY plane, and the velocity components $v_{X}$ and $v_{Y}$, for each measured track.

### Magnetic Transport Measurements.

The following sections present results of magnetic transport for all of the samples measured, shown in Table 1. The experimental setup is first validated by transporting commercially available, diamagnetic polystyrene (PS) microspheres (5 μm diameter) in paramagnetic solution ($0.6\;M\;{MnCl}_{2}$). Following that, measurements are taken for various samples of diamagnetic HOPG microflakes in diamagnetic solutions: uncoated HOPG microflakes in an acetone–water mixture and POPC lipid-coated HOPG microflakes in 20 mM NaCl aqueous solution. For each sample, three to five particles and a total of 11–12 tracks are measured.

**Table 1.** Samples of diamagnetic microparticles in solution for magnetic transport experiments

Sample	Particle	Solution	$\left(\chi_{2}-\chi_{1}\right)$	$N_{p}$	$N_{t}$
A	PS beads 5 μm ∅	Paramagnetic $0.6\;M\;{MnCl}_{2}$ aqueous solution	$-1.12\times 10^{-4}$	1	10
B	HOPG	40−60% acetone–water	$-7.4\times 10^{-5}$	3	12
C	Lipid-coated HOPG	20 mM NaCl aqueous solution	$-7.3\times 10^{-5}$	5	11

$\left(\chi_{2}-\chi_{1}\right)$, difference in volume magnetic susceptibility between particle and solution; $N_{p}$, no. of particles measured; $N_{t}$, total no. of tracks measured.

### Magnetic Transport of Polystyrene Microspheres in Paramagnetic Solution.

Like graphite, PS is diamagnetic, but more weakly so, with an isotropic volume magnetic susceptibility ($\chi_{PS}=-8.2\times 10^{-6}$) (23) very close to that of water ($\chi_{water}=-9.0\times 10^{-6}$). This makes magnetic manipulation of PS in water very difficult for microspheres of small volume owing to the very small magnetic susceptibility contrast between particle and fluid. This susceptibility contrast can be enhanced using a paramagnetic salt aqueous solution with positive magnetic susceptibility (that increases with increasing paramagnetic salt concentration) to exert useful magnetic forces on the PS microspheres (23, 62–64). Here, we use a 0.6-M ${MnCl}_{2}$ aqueous solution with $\chi_{1,{MnCl}_{2}}=1.04\times 10^{-4}$ (65) that results in a particle–fluid susceptibility contrast similar to that of HOPG in aqueous solutions [see $\left(\chi_{2}-\chi_{1}\right)$ in Table 1]. To reduce sticking of the beads to the capillary walls, nonionic surfactant is added at a concentration (∼3 mM) that does not modify the viscosity of the original solution (*Materials and Methods*).

**Fig. 2.**
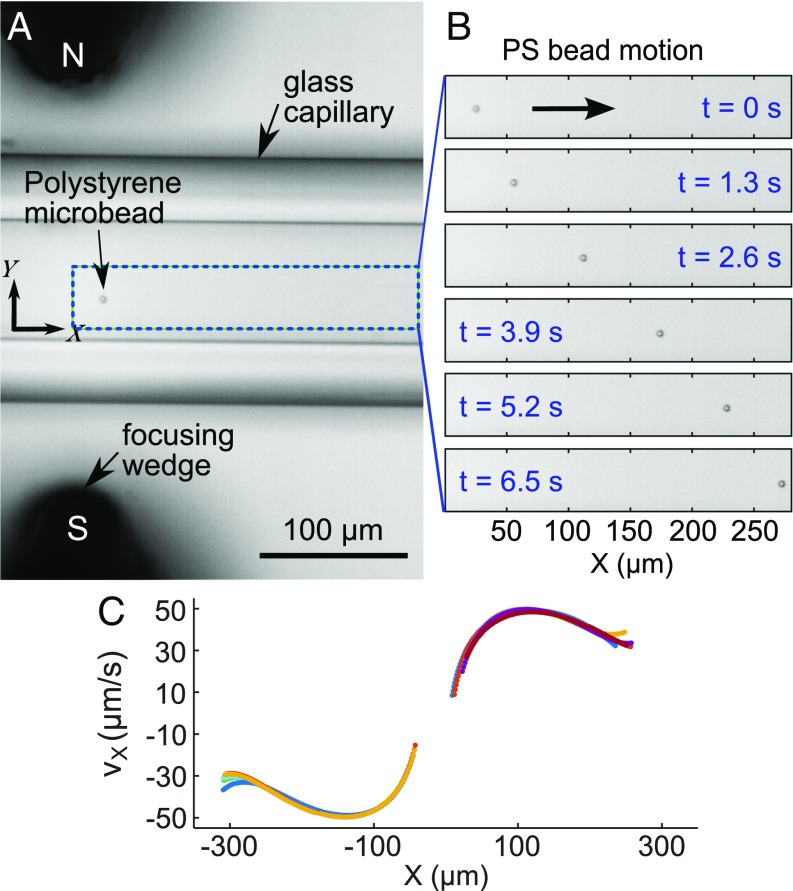
Magnetic transport of 5-μm-diameter polystyrene bead in $0.6\;M\;{MnCl}_{2}$ aqueous solution. (*A*) Brightfield image of individual microbead transported in capillary between magnets and focusing wedges. (*B*) Time sequence of bead-motion images (every 1.3 s). (*C*) Measured particle velocities vs. position X for all 10 tracks.

Fig. 2 *A* and *B* shows a time sequence of images of the magnetic transport of a single PS microsphere. The particle travels approximately in a straight line along X, away from the center of the focusing wedges, with negligible motion along Y. Fig. 2*C* shows the particle velocities obtained by single-particle tracking for 10 different tracks, which show good reproducibility. The measured variation in velocity along each track reflects changes in $B_{0}\nabla B_{0}$ (Eq. **5**). Motion over distances ∼250 μm to either side of X=0 is shown, with peak velocities up to ∼50 μm/s. *SI Appendix*, Movie S1 shows PS bead transport. We have found one previous study of label-free magnetophoretic transport of PS microspheres in absence of fluid flow (in a dispersion of superparamagnetic nanoparticles to enhance magnetic contrast) that reported particle velocities ∼2 μm/s supported by a theoretical description (25).

To compare our measured velocities (Fig. 2*C*) with the predicted theoretical ones, we calculate their ratio, $v_{X,rel}$. This yields a mean ratio $v_{X,rel}=0.18\pm 0.02$ for all PS tracks (see *Materials and Methods* for details on error bars). We calculate the theoretical magnetophoretic velocities using Eq. **6** for magnetically isotropic microspheres (*Materials and Methods*). The value of $B_{0}{\left(\nabla B_{0}\right)}_{X}$ at each particle position along a measured transport track is obtained from magnetic-field simulations (*Materials and Methods*).

The measured reduction in velocity by a factor of ∼5.6 compared with theory is due to the proximity of the PS microspheres to the capillary surface. The microspheres fall under gravity with a Stokes sedimentation velocity along Z (*Materials and Methods*) of ∼0.6 μm/s so that it takes ∼1 min for a bead to fall over the half width of the capillary toward the bottom wall. This time is shorter than the experiment-preparation time and hence proximity to the capillary surface is hard to avoid. While the sliding friction force for particle motion in contact with the capillary surface is negligible compared with the magnetic force acting on the microspheres (*Materials and Methods*), this proximity gives rise to increased viscous drag for particle motion near the capillary wall, as well as to particle–surface interactions. The increase in viscous drag is due to the difficulty for the particle to displace the fluid as it moves close to the wall. It is well known that for a sphere of radius r moving parallel to a plane wall at a wall-to-bead-center distance h, with a wall–bead gap δ=h−r, the translational viscous drag coefficient increases as δ/r decreases, up to ∼3−3.5 times for very small gaps δ/r ∼ 0.01. This has been shown both theoretically [Faxén’s approximation (66, 67) and O’Neill’s full numerical solution (68)] and experimentally (69). This increase in viscous drag would hence cause a reduction in particle velocity by the same factor ∼3−3.5 at most. As we measure a velocity reduction compared with theory by a factor larger than this, it is apparent that significant particle–surface interactions further slow down particle motion. This is not surprising as PS beads in aqueous solution tend to stick to glass (70, 71), particularly in the presence of a relatively high salt concentration as is the case here (despite the use of surfactant). PS beads are typically slightly negatively charged in water at neutral pH (negative zeta potential) (72–74) and so are glass surfaces (owing to the dissociation of terminal silanol groups) (75). In salt solution, dissolved ions give rise to electrical double layers around charged surfaces, significantly screening the long-range electrostatic repulsion between like charges and allowing short-range attractive van der Waals forces to lead to sticking (75–78). Long surfactant molecules bind the hydrophobic PS bead surface (and glass), reducing attractive interactions via steric hindrance. We estimate that the surface-interaction forces (*Materials and Methods*) for our moving microspheres are ∼5 pN on average, compared with viscous drag forces ∼7 pN (using $∼3\gamma_{sph}$) and magnetic forces ∼12 pN.

### Magnetic Transport of Graphitic Microflakes.

**Fig. 3.**
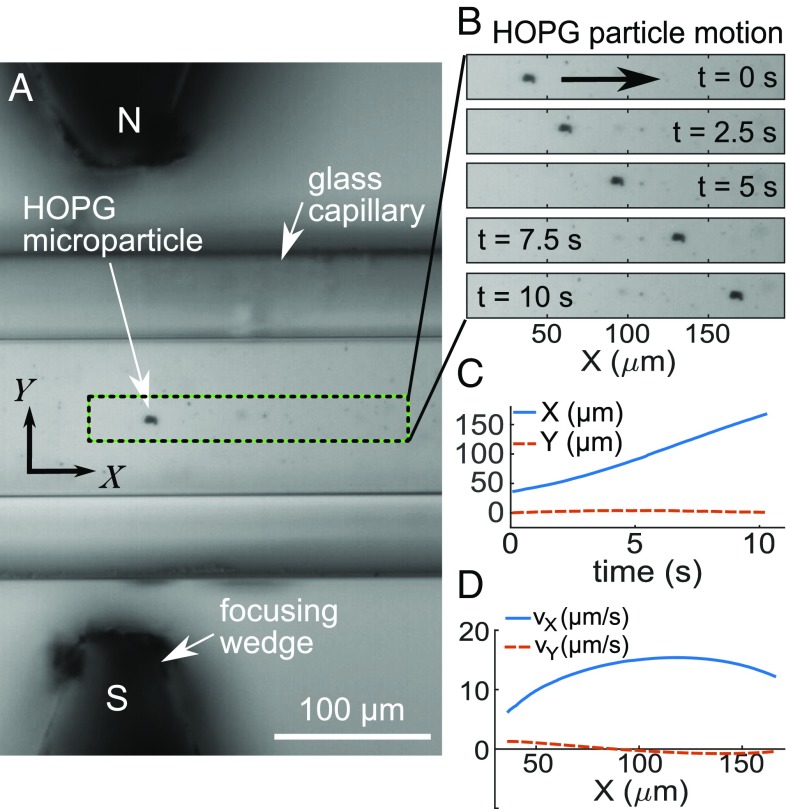
Magnetic transport of a graphitic HOPG microflake (Table 1, sample B). (*A*) Brightfield image of microflake in capillary between focusing iron wedges. (*B*) Sequence of movie frames (every 2.5 s) during transport. (*C*) Corresponding particle positions X and Y as a function of time. (*D*) Corresponding particle velocities along X and Y.

We now present results for the magnetic transport of uncoated HOPG microflakes ($\chi_{∥}=-8.2\times 10^{-5}$) in an acetone–water mixture ($\chi_{mixture}=-7.9\times 10^{-6}$) (*Materials and Methods*) corresponding to sample B in Table 1. Fig. 3 *A* and *B* shows image data for the motion of a single microflake and Fig. 3 *C* and *D* shows the tracked particle positions and velocities, respectively. The particle covers a distance along X of ∼130 μm with a maximum velocity ∼15 μm/s (*SI Appendix*, Movie S2).

**Fig. 4.**
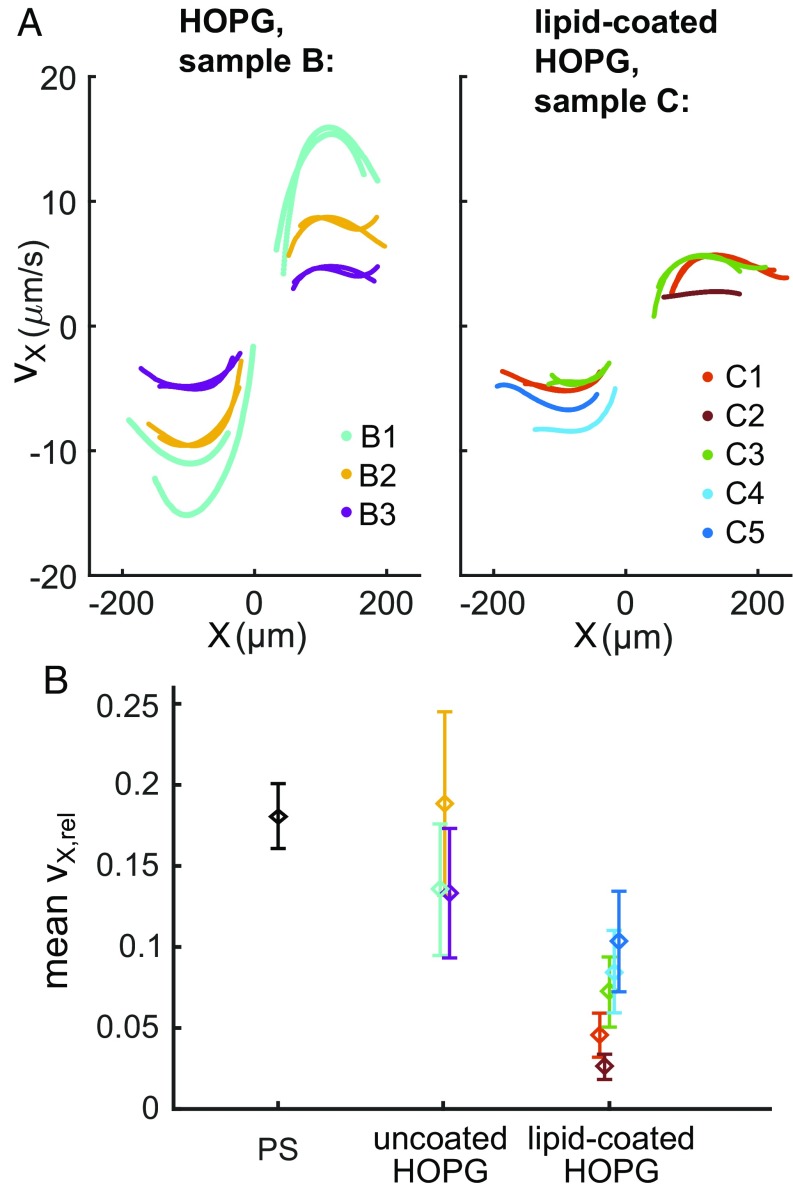
(*A*) Measured magnetophoretic velocities vs. position X for all tracks and all individual HOPG microflakes [B1–B3 for sample B (*Left*) and C1–C5 for sample C (*Right*)] (*SI Appendix*, Note 2), for transport to either side of X=0. Each color corresponds to a different microflake. (*B*) Mean ratio $v_{X,rel}$ of experimental to predicted velocity for all particles and sample types (see *Materials and Methods* for details on error bars).

Fig. 4*A*, *Left* shows the measured velocities vs. position X for all individual microflakes tracked for sample B (Table 1). These demonstrate particle motion over relatively long length scales up to 200 μm to either side of the center of the wedges (X=0). Peak velocities measured range between a few micrometers per second and ∼15 μm/s. The calculated magnetic forces acting on the microflakes are ∼1−5 pN. The spread of velocities in Fig. 4*A* is mostly due to differences in particle volume (*SI Appendix*, Note 2), with tracks being mostly reproducible for each individual particle (shown in the same color).

The velocities measured for the uncoated HOPG particles (sample B) are lower than the predicted ones by a factor of 5–8, as shown in Fig. 4*B*, that plots the mean ratio of experimental to predicted velocity, $v_{X,rel}$, for each particle, for all samples in Table 1 (*Materials and Methods*, *Theoretical Velocity Calculations*). Similarly to PS beads, HOPG microflakes are slowed down by their proximity to the capillary wall. As the particles are twice as dense as their corresponding fluid, they sediment fast in ∼10 s, a time scale shorter than the experiment-preparation time [sedimentation velocity ∼4 μm/s (*Materials and Methods*)]. Consequently, transport velocity is reduced by an increase in viscous drag close to the capillary wall by a factor that, for disk-shaped particles, can be significantly larger than the factor of ∼3 for spheres (79). While uncoated HOPG particles in the nonsaline acetone–water mixture stick somewhat less to the bottom capillary wall than PS particles, particle–surface interactions likely also slow down particle motion (owing to large microflake–glass contact areas).

In absolute terms, the measured velocities for HOPG microflakes (Fig. 4*A*) are lower than those for PS microspheres (Fig. 2*C*) due to the smaller particle volume for the HOPG microflakes ($6-32\;\mu m^{3}$) compared with the PS microspheres ($65\;\mu m^{3}$), to the lower magnetic susceptibility contrast $\left(\chi_{2}-\chi_{1}\right)$ for HOPG in diamagnetic solutions (Table 1), and to the abovementioned effects.

### Fully Biocompatible Transport of Graphitic Microflakes.

Having demonstrated contactless magnetic motion of graphitic microparticles in a diamagnetic acetone–water solution, we go on to demonstrate magnetic transport in a fully biocompatible system. For this purpose, we use lipid-coated HOPG microflakes in diamagnetic NaCl aqueous solution ($\chi_{1}\approx \chi_{water}=-9.0\times 10^{-6}$) (Table 1, sample C). Data for these particles are presented in Fig. 4 (*SI Appendix*, Movie S3). Particle transport velocities are slower compared with those for uncoated microflakes (Fig. 4*A*) and measured velocities are lower than the predicted ones by at least one order of magnitude (Fig. 4*B*). This is due to significant interactions between the lipid coating and negatively charged glass in the ionic salt solution [POPC lipids have polar head groups and can be bound by electrolyte and water ions (74, 80, 81)]. Electrical double-layer and structured water-layer effects (denser solvent packing and increased effective viscosity near charged surfaces) might also play a role (82, 83).

These results demonstrate the validity of our magnetic transport method in biocompatible environments and biologically relevant contexts, opening up possibilities for interesting applications. For instance, HOPG microflakes could be coated with fluorescently labeled lipids or with biotinylated lipids for subsequent conjugation to biomolecules of choice via streptavidin, with such particle functionalizations enabling unexplored lab-on-a-chip assays and applications.

## Discussion and Conclusions

We have presented an important demonstration of label-free, contactless magnetic transport of diamagnetic graphitic microparticles in diamagnetic aqueous solutions. Our results prove that micrometer-sized graphite can be magnetically manipulated and that diamagnetic responses can indeed be strong enough to generate useful manipulation in solution.

We have presented a theoretical model of magnetic transport of magnetically anisotropic diamagnetic microparticles in solution which captures the essential physics of the process. Using this model, we have designed an experimental configuration that provides an inhomogeneous magnetic-field landscape with a suitable magnetic-field gradient to achieve directed transport. We have shown experimentally that transport works as described in our model by magnetically transporting and tracking the motion of submerged HOPG microflakes over distances ∼200 μm. We have presented data for both uncoated HOPG in a water–acetone mixture and lipid-coated HOPG in NaCl aqueous solution, measuring velocities up to ∼15 μm/s. These velocities are 10 times higher than those previously reported for diamagnetic cells (12−16 μm in diameter) in diamagnetic solutions (14). Additionally, our results for lipid-coated HOPG in NaCl aqueous solution prove that magnetic transport of graphitic microparticles is possible in a fully biocompatible context, establishing the basis for multiple applications in biological physics/chemistry/medicine.

Comparison between theoretical model predictions and measured particle velocities reveals that particle motion is slowed down by the presence of significant particle–surface interactions, particularly for lipid-coated HOPG microflakes. The effect of these interactions is not included in our model. Nonetheless, our model and experimental observations allow the quantitative estimation of these additional interactions and show that magnetic transport is possible in the presence of particle–wall interaction forces of a similar order of magnitude to magnetic transport forces. Our techniques can provide a tool to characterize lipid–glass interactions and lipid–water interfaces in different experimental conditions of interest to biological applications.

Particle–surface interactions could be reduced via surface passivation (e.g., pegylation of glass) or interactions could be avoided altogether by generating 2D guides for particle transport (via nontrivial electrical or magnetic schemes) to avoid sedimentation and ensure particles are suspended away from the surface. Both approaches would result in higher HOPG magnetophoretic velocities.

As future steps, the possibility of generating 3D magnetic traps for graphitic microparticles in solution is interesting for applications such as 3D force sensing. HOPG microflakes can also be oriented and rotationally trapped in solution and hence used for applying or sensing torque and rotary motion in microscopic systems in fluid (30). As HOPG is also strongly electrically conducting and polarizable along its in-plane direction, transport and trapping schemes in inhomogeneous fields can also be devised for electrical manipulation. Combinations of magnetic and electric fields can allow advanced microparticle manipulation schemes that combine orientation, translation, and trapping for biophysical/biochemical studies. Given that graphitic particles are biocompatible, can be solubilized and functionalized, and can be used as biological cell substrates in physiological solutions, the results presented here are of interest to a wide range of applications based on lab-on-a-chip and microfluidic devices, as well as to tissue-engineering applications. Combined magnetophoresis and electrical impedance detection schemes such as previously reported for the separation of circulating tumor cells (11) could also be devised. Photothermal temperature-jump studies would also be possible, given the ability of HOPG to absorb visible and infrared light and heat up (35, 36, 39, 84). Full contactless control of microparticle position would improve any such applications and could potentially reduce the need for microfluidic flow control.

The magnetic transport principles presented here also apply to smaller graphitic particles such as graphene platelets or carbon nanotubes. These could be magnetically transported by generating large enough magnetic fields and field gradients to compensate for the reduction in magnetic force due to the smaller particle volume compared with the micrometer-sized HOPG particles in this study. A route with great potential would be exploring the use of microfabricated wires and permanent magnets for on-chip magnetic transport schemes, as scaling down components significantly increases magnetic-field gradients. Additionally, magnetic transport schemes would also be possible in air or vacuum and substantially easier to achieve than in fluid, given the reduction in viscous drag forces in gas compared with liquid.

## Materials and Methods

### Sample Preparation.

We use HOPG (part no. AGG3045-1010 from Agar Scientific; 0.4°±0.1° mosaic spread, impurity level ≤10 ppm). Graphitic microparticles can be stably dispersed in a mixture of water and acetone with volume fractions 60% and 40%, respectively (59). This enables easy preparation of HOPG dispersions. Small HOPG pieces ($∼1\;{mm}^{3}$) extracted with tweezers from the bulk are broken down into micrometer-sized flakes by low-power bath sonication in the acetone–water mixture at temperatures <30 °C, for a sonication time of 1 h. Longer sonication times can be used to obtain smaller particle sizes and a higher number of particles. Alternatively, we generate dispersions of POPC lipid-coated HOPG microflakes in 20 mM NaCl aqueous solution via bath sonication as detailed previously (30). The low final concentration of lipids in solution (0.6 mg/ml) does not alter the solution viscosity according to the viscosity law for noninteracting mixtures (85) [the molar fraction of lipids is negligible (POPC molecular weight: 760 g/mol)]. The dimensions of the microflakes used in experiments and their uncertainties are given in *SI Appendix*, Note 2.

Dilute dispersions of polystyrene microspheres (5 μm diameter; part no. PPS-5.0, Kisker Biotech) are prepared in paramagnetic 0.6 M ${MnCl}_{2}$ aqueous solution. Nonionic surfactant [Triton X-100, 2 μl added to 1 ml of the solution (∼3 mM final concentration)] is added to reduce sticking of beads to the capillary wall. The uncertainty in the microsphere radius is 5% as the beads are monodisperse only to this level.

We prepare very dilute dispersions for the manipulation of individual microparticles and fill thin glass capillaries with them. The capillary ends are sealed with nail varnish to prevent fluid evaporation and flow.

### SQUID Magnetometry of HOPG.

A magnetic property measurement system (MPMS-5S; Quantum Design Inc.) was used to acquire SQUID data of magnetization vs. magnetic field for bulk HOPG (3 mm×2 mm×1 mm piece) to measure its in-plane and out-of-plane volume magnetic susceptibility components (*SI Appendix*, Note 1).

### Raman Spectroscopy of HOPG.

Raman spectra for bulk HOPG and for individual HOPG microflakes were collected for visible light excitation (488 nm, <2.5 mW, ∼3 μm Gaussian width at the sample). Several spectra were acquired for each sample over a square grid with 2-μm spacings and subsequently averaged. Both samples clearly presented a G peak at $∼1,580\;{cm}^{-1}$, characteristic of pristine HOPG (or single-crystal graphite). An increased level of disorder was measured for the microflakes compared with bulk HOPG, evidenced by the appearance of a D peak at $∼1,350\;{cm}^{-1}$ and by increased ratios of D-peak intensity to G-peak intensity (*SI Appendix*, Note 3) (55–58, 86).

### Magnetophoretic Velocity for Magnetically Isotropic Microspheres.

In the case of magnetically isotropic spherical particles, following from the theory, the expression for the magnetophoretic velocity is

$$v_{k}=\frac{V_{sph}}{\mu_{0}\gamma_{sph}}\left(\chi_{2}-\chi_{1}\right)B_{0}{\left(\nabla B_{0}\right)}_{k}\;,$$ [6]

where $\chi_{2}$ is the isotropic magnetic susceptibility of the microspheres, $V_{sph}=4\pi r^{3}/3$ is the volume of the spheres of radius r, and $\gamma_{sph}$ is the Stokes viscous drag coefficient for spheres (see *Viscous Drag Coefficient*). For experiments with 5-μm-diameter PS microspheres in 0.6 M ${MnCl}_{2}$ aqueous solution, the magnetic susceptibility for the particles is $\chi_{2,PS}=-8.2\times 10^{-6}$ (23) and that for the solution is $\chi_{1,{MnCl}_{2}}=1.04\times 10^{-4}$ (65).

### Viscous Drag Coefficient.

For spherical microparticles, the viscous drag coefficient for translational motion in any direction is given by the Stokes formula $\gamma_{sph}=6\pi \eta r$, where η is the dynamic viscosity of the solution and r is the microsphere radius. For experiments with PS microspheres in 0.6 M ${MnCl}_{2}$ aqueous solution, the viscosity of the solution at 25 ° C is $1.1\times 10^{-3}\;Pa.s$ (87). This viscosity is not affected by the small concentration of Triton X-100 surfactant [viscosity 0.24 Pa.s, molecular weight 625 g/mol (88)] added to the solution (∼3 mM final concentration), as the molar fraction of surfactant is negligible when considering the noninteracting law for viscosity of mixtures (85).

By approximating our HOPG microflakes as elliptical disks with surface ellipse semiaxes a and b (along x and y, respectively) and disk half-thickness c (along z) (Fig. 1*B*), we can use an extension of Stokes law to calculate the translational viscous drag coefficients, $\gamma_{k}$ (k=x,y,z), along each direction of motion for the microflakes. This method has been used to calculate coefficients for prisms or cylinders with an error <4% compared with corresponding measurements (89). The method consists of applying a shape factor to the drag coefficient of the equivalent sphere, with the shape factor depending on both the cross-sectional area of the object in the direction of motion and the total effective surface of the object. In our work, the relevant viscous drag coefficients are $\gamma_{x}$ and $\gamma_{y}$, as the HOPG microflakes move along the direction of their a or b semiaxes (or a mixture of both; *SI Appendix*, Note 2). These drag coefficients are given by

$$\gamma_{k}=\gamma_{0,k}S_{k}\;,$$ [7]

where k=x,y and $\gamma_{0,k}=3\pi \eta d_{c,k}$ is the Stokes drag coefficient for an equivalent sphere with the same cross-section as that of the elliptical disk normal to its direction of motion ($d_{c,k}$ is the diameter of such equivalent sphere). $S_{k}$ is the shape factor for motion along k, given by

$$S_{k}=\frac{1}{3}+\frac{2}{3}\frac{d_{s}}{d_{c,k}}\;,$$ [8]

where $d_{s}$ is the diameter of a sphere whose surface is equal to that of the elliptical disk (89).

The dynamic viscosities of water and pure acetone at 25 °C are, respectively, $\eta_{water}\;∼\;8.9\times 10^{-4}\;Pa.s$ and $\eta_{acetone}\;∼\;3\times 10^{-4}\;Pa.s$ (90). The dynamic viscosity of our acetone–water mixture (40% acetone volume fraction, equivalent to 34% acetone weight fraction and to 14% acetone molar fraction) is obtained by interpolating previously reported data for interacting mixtures with various molar fractions (90) and is $\eta_{mixture}\;∼\;1.4\times 10^{-3}\;Pa.s$. The dynamic viscosity of the 20-mM NaCl aqueous solution is approximately the same as that of water (negligible molar fraction of NaCl). Considering no uncertainty in the value of the fluid viscosity, calculated values for the viscous drag coefficients for HOPG microflakes have relative uncertainties 4−8%, owing to uncertainty in the determination of microflake size (*SI Appendix*, Note 2).

### Equation of Motion.

The equation of motion for the translation of the particle along x is given by

$$m\ddot{x}=-\gamma_{x}\dot{x}+F_{m,x}+\sqrt{2k_{B}T\gamma_{x}}W\left(t\right)\;,$$ [9]

where m is the mass of the particle, $\ddot{x}$ and $\dot{x}$ are the particle’s acceleration and velocity along x, respectively, $\gamma_{x}$ is the viscous drag coefficient for particle motion along x, and $F_{m,x}$ is the external magnetic force acting along x. The last term corresponds to the stochastic thermal Brownian fluctuations at temperature T in solution, where $k_{B}$ is Boltzmann’s constant and W(t) is a normally distributed random variable.

For micrometer-sized particles in fluid, inertial forces ($m\ddot{x}$) can be neglected in the equation of motion in comparison with viscous forces ($-\gamma_{x}\dot{x}$) (low Reynolds number regime), as the characteristic time, $m/\gamma_{x}$, in which translational motion is damped by friction is very short (≤μs) and velocity changes can be considered instantaneous over observable time scales. The effect of Brownian fluctuations can also be neglected given that we operate in the high–Peclet-number regime (91) where the time scale of directed advective motion ($t_{v}=L/v$, over length scales L∼1−200 μm and velocities v∼1−20 μm/s) is much faster than the time scale of Brownian diffusive motion ($t_{d}=L^{2}/D$, where $D=k_{B}T/\gamma$ is the diffusion coefficient at temperature T and γ is the appropriate viscous drag coefficient).

### Sliding Friction Force.

The sliding friction force, $F_{f}$, for particle motion parallel to and in contact with a surface is given by Coulomb’s approximation $F_{f}=\mu F_{n}=\mu \left(\rho_{2}-\rho_{1}\right)Vg$, where μ is the kinetic sliding friction coefficient and $F_{n}$ is the force normal to the surface, here resulting from the difference of gravitational and buoyant forces (g is the acceleration of gravity, V is the particle volume, and $\rho_{2}$ and $\rho_{1}$ are the densities of particle and fluid, respectively).

The density of the particle ($\rho_{2}$) is either $\rho_{PS}=1.05\times 10^{3}\;{kg/m}^{3}$ for polystyrene beads (sample A in Table 1) or $\rho_{HOPG}=2.24\times 10^{3}\;{kg/m}^{3}$ for HOPG (samples B and C in Table 1). The density of the solution ($\rho_{1}$) is either $\rho_{acewater}\;∼\;0.94\times 10^{3}\;{kg/m}^{3}$ for the acetone–water mixture (sample B in Table 1) or $\rho_{water}=0.997\times 10^{3}\;{kg/m}^{3}$ for the NaCl aqueous solution (sample C in Table 1) and the paramagnetic salt aqueous solution (sample A in Table 1).

For PS microspheres, the sliding friction force (using μ≤1) is of order $\leq 10^{-2}\;pN$, at least three orders of magnitude smaller than the magnetic force acting on the beads (∼12 pN). For HOPG microflakes, the sliding friction force (for μ≤1) is of order $\leq 10^{-1}\;pN$, at least one order of magnitude smaller than the magnetic force acting on the microflakes (a few piconewtons). The sliding friction force is neglected in the calculation of theoretical velocities as μ is likely significantly smaller than 1 for lubricated friction. Note that the sliding friction force does not account for possible particle–surface interactions, such as electrostatic ones.

### Estimation of Particle–Surface Interaction Forces.

The force on a microsphere arising from particle–surface interactions is calculated as $F_{int,x}=3\gamma_{sph}\;v_{exp,x}-F_{m,x}$, where it is assumed that the viscous drag coefficient is increased by a factor of ∼3 for motion close to the capillary wall, $v_{exp,x}$ is the measured particle velocity, and $F_{m,x}=\frac{V}{\mu_{0}}\left(\chi_{2}-\chi_{1}\right)B_{0}{\left(\nabla B_{0}\right)}_{x}$ is the applied magnetic transport force.

### Magnetic Field Modeling with Radia.

The magnetic field is simulated using the Radia package for Mathematica for semianalytical, 3D magnetostatics modeling (61). The NdFeB magnet blocks are modeled with a remanent magnetization of 1.4 T (as reported by the manufacturer). The steel wedges are assigned nonlinear ferromagnetic properties: a volume magnetic susceptibility of 34 and a saturation magnetization of 2 T. These parameters are determined by optimizing agreement of the measured and simulated data for a single block magnet and wedge (*SI Appendix*, Note 5). The steel wedge geometry is subdivided into a grid with 25×25×25 sections to account for inhomogeneities of the field inside the material (as recommended in Radia instructions). The magnetic-field simulations for two block magnets and two wedges (Fig. 1 *A* and *C*) are carried out with the abovementioned optimized parameters to obtain maps of $B_{0}{\left(\nabla B_{0}\right)}_{X}$ and $B_{0}{\left(\nabla B_{0}\right)}_{Y}$ vs. X and Y (Fig. 1 *D* and *E*). We estimate that the uncertainty in the calculated $B_{0}\left(\nabla B_{0}\right)$ values (due to small misalignments and/or tilts with respect to the experimental setup) is up to ∼10%.

### Theoretical Velocity Calculations.

The theoretical velocities of the HOPG microflakes in their tracks can be calculated with Eq. **5**. Eq. **5** is defined in the particle frame of reference. However, since the HOPG microflakes orient with their planes parallel to the field-line direction (30) (approximately parallel to Y in experiments) and typically stay parallel to the XY plane as they move (this is their preferential stable orientation due to their flake-like shape), we have that x∥X and y∥Y. Therefore, we can use Eq. **5** to calculate theoretical velocities $v_{X}$ and $v_{Y}$ in the laboratory frame of reference and compare with the measured velocities.

The particle volume V is calculated assuming that the microflakes are elliptical disks (V=2πabc), with their dimensions measured from the acquired microscope images via image processing (*Image Processing and Single-Particle Tracking*). The viscous drag coefficient $\gamma_{k}$ is calculated using the expressions for elliptical disks and the dynamic viscosity of the fluid as explained in *Viscous Drag Coefficient*. The volume magnetic susceptibility measured for HOPG via SQUID ($\chi_{∥}$) is used. The magnetic properties of the solutions used are as follows. The magnetic susceptibility of the acetone–water mixture is calculated according to the additive law for mixtures (assuming no interaction between components) (92, 93), $\chi_{mixture}=w_{water}\chi_{water}+w_{acetone}\chi_{acetone}$, where $w_{water}=0.66$ and $w_{acetone}=0.34$ are the weight fractions of water and acetone corresponding to the 60% and 40% volume mixture, respectively, and with $\chi_{water}=-9.0\times 10^{-6}$ and $\chi_{acetone}=-5.8\times 10^{-6}$ (94). The result is $\chi_{mixture}=-7.9\times 10^{-6}$. The volume magnetic susceptibility of the 20-mM NaCl aqueous solution (weakly diamagnetic) is assumed to be the same as that of water. Finally, the magnetic field and field gradient are obtained from 3D simulations with Radia (*Magnetic Field Modeling with Radia*), as presented in Fig. 1. Simulations are needed as the small gap between the iron wedges precludes insertion of a standard Gaussmeter probe. The simulations generate maps of $B_{0}\left(\nabla B_{0}\right)$ values as a function of position on the XY plane. These maps are used to generate maps of theoretical velocity values. The instantaneous theoretical velocities for a given measured particle trajectory on the XY plane are then obtained via interpolation of the previous velocity maps for each point in the measured trajectory. Mean values of $v_{X,rel}$ (ratio of experimental to predicted theoretical velocity) are calculated by averaging over all points in a track and over all tracks for a given particle. The use of $v_{X,rel}$ allows comparison of results for different sample types by normalizing to account for the varying sizes and shapes of the particles measured (larger particles move faster), for the size-dependent viscous drag coefficient, for the different magnetic properties of solutions and particles, for differences in medium viscosity, and for changes in magnetic field and field gradient along the particle track.

The overall uncertainty in the $v_{X,rel}$ values for each HOPG microflake is ∼30%. This value results from combining the random error of the various tracks measured for each microflake (1−13%) with the relative uncertainty of each $v_{X,rel}$ value (∼29%). The latter is due to the propagation of ∼2% uncertainty in measured particle velocities, 25−27% uncertainty in microflake volume estimation, 4−8% uncertainty in calculated viscous drag coefficient, ∼1% uncertainty in measured HOPG magnetic susceptibility, and ∼10% uncertainty in simulated $B_{0}{\left(\nabla B_{0}\right)}_{X}$ values.

For PS microbeads, the theoretical velocity is calculated in a similar way but using Eq. **6** instead of Eq. **5**. Calculated $v_{X,rel}$ values for PS microspheres have relative uncertainties ∼14%, with the main contributions being the uncertainty in $B_{0}{\left(\nabla B_{0}\right)}_{X}$ (∼10%) and the uncertainty in the microsphere radius (∼5%).

### Image Processing and Single-Particle Tracking.

Image-processing single-particle–tracking algorithms custom written in Matlab are used to extract the microparticle position and dimensions from the acquired transport image sequences. Particles are approximated as elliptical disks as shown in Fig. 1*B*. As particles move along the direction of their a or b axis, images of their approximately elliptical surface area are captured on video. The position of the particle on each frame on the video sequence is first automatically detected via morphological operations and intensity thresholding to generate a binary mask for the particle. The ellipse that best fits the shape of this mask is then obtained. The center of mass of the fitted ellipse yields the position of the particle with an accuracy below 20 nm (assessed by tracking synthetic images of microparticles generated at known positions with controlled background noise and particle edge roughness and eccentricity). Positions corresponding to the same particle on different frames are then linked into a track. In this way, particle position as a function of time, and velocity, can be analyzed. The relative uncertainty in particle displacement (between frames) and in measured particle velocity is ∼2%. Additionally, the half-lengths of the major and minor axes of the ellipse are obtained (averaging over results for all frames in a track) to determine the particle size (*SI Appendix*, Note 2).

### Stokes Sedimentation Velocity.

The Stokes sedimentation velocity for a sphere of radius r can be calculated from the balance of the gravitational, buoyant, and viscous drag forces as $v_{sedim}=2gr^{2}\left(\rho_{2}-\rho_{1}\right)/\left(9\eta \right)$. Brownian motion is neglected in the calculation as the Péclet number is sufficiently high also for vertical motion under gravity. This calculation assumes that the particle is a sphere. As an approximation for the HOPG microflakes, an equivalent sphere is used that has the same volume as a typical microflake of average size (*SI Appendix*, Note 2).

The calculated Stokes sedimentation velocities for PS microspheres and HOPG microflakes (main text) show that particles move very close to the capillary surface. Indeed, it is the case that particles (PS or HOPG) that get stuck to the bottom capillary wall appear in focus on the acquired images on which a moving particle is also in focus (the objective’s depth of field along Z is 8.5 μm).

## Supplementary Material

Supplementary File

Supplementary File

Supplementary File

Supplementary File
