# Supplementary material for: Magnetic control of graphitic microparticles in aqueous solutions
Source: Proc Natl Acad Sci U S A. 2019 Jan 25;116(7):2425–34. doi: 10.1073/pnas.1817989116 (PMC6377480; doi:10.1073/pnas.1817989116)
Supplement: Supplementary File [file pnas.1817989116.sapp.pdf]

Supplementary Information for:
Magnetic control of graphitic microparticles in aqueous solutions

J. Nguyen, D. V. Conca, J. Stein, L. Bovo, C. A. Howard, and I. Llorente García

SUPPLEMENTARY VIDEOS

Supplementary video 1.
Video file *particle_PS.mp4*: Magnetic transport of a $5\,\mu$m-diameter, diamagnetic polystyrene microsphere in a paramagnetic 0.6 M $MnCl_2$ aqueous solution. The particle moves inside a capillary ($100\,\mu$m inner width) along the horizontal field gradient generated by two NdFeB permanent magnet blocks and two focusing iron wedges. The particle is magnetically transported over a horizontal distance $\sim 250\,\mu$m with a maximum velocity $\sim 50\,\mu$m/s as shown by the data in Fig. 2 (main text).

Supplementary video 2.
Video file *particle_B1_HOPG.mp4*: Magnetic transport of an uncoated graphite microflake in a diamagnetic acetone-water mixture (40% acetone, 60% water volume fractions). The video shows a sequence of real-time microscopy images where a HOPG microflake is transported along a horizontal magnetic field gradient inside a capillary ($100\,\mu$m inner width) placed between two NdFeB permanent magnet blocks and focusing iron wedges. The video corresponds to particle B1 with lateral half-sizes $\sim 4\,\mu\text{m} \times 2\,\mu\text{m}$ (Supplementary Table S2). The particle is magnetically transported over a horizontal distance of $\sim 130\,\mu$m with a maximum velocity $\sim 15\,\mu$m/s as shown by the data in Fig. 3 (main text).

Supplementary video 3.
Video file *particle_C1_lipid_coated_HOPG.mp4*: Fully biocompatible magnetic transport of a POPC lipid-coated graphite microflake in diamagnetic 20 mM NaCl aqueous solution. The video shows a real-time image sequence for the transport of the coated HOPG particle along a horizontal magnetic field gradient in the same experimental setup as for the previous video file. The video corresponds to particle C1 with lateral half-sizes $\sim 3\,\mu\text{m} \times 2\,\mu\text{m}$ (Supplementary Table S2).

SUPPLEMENTARY NOTE 1: DETAILS OF SQUID MAGNETOMETRY OF HOPG

We used a superconducting quantum interference device (SQUID) to measure the dimensionless volume magnetic susceptibilities components (SI units) of a piece ($3\,\text{mm} \times 2\,\text{mm} \times 1\,\text{mm}$) of the HOPG bulk used to prepare microflakes. Note that measuring the magnetisation of a single HOPG microflake is beyond the sensitivity of SQUID. We used a Magnetic Property Measurement System (MPMS-5S by Quantum Design Inc.) to acquire the SQUID data. Figure S1a shows the measured magnetisation versus magnetic field for bulk HOPG, used to obtain the in-plane and out-of-plane volume magnetic susceptibility components. The main linear diamagnetic backgrounds from the measured magnetisation vs magnetic field at 295 K yield $\chi_\perp = (-5.82 \pm 0.01) \times 10^{-4}$ and $\chi_\parallel = (-8.2 \pm 0.1) \times 10^{-5}$. These values are within the range reported in the literature (Table S1).

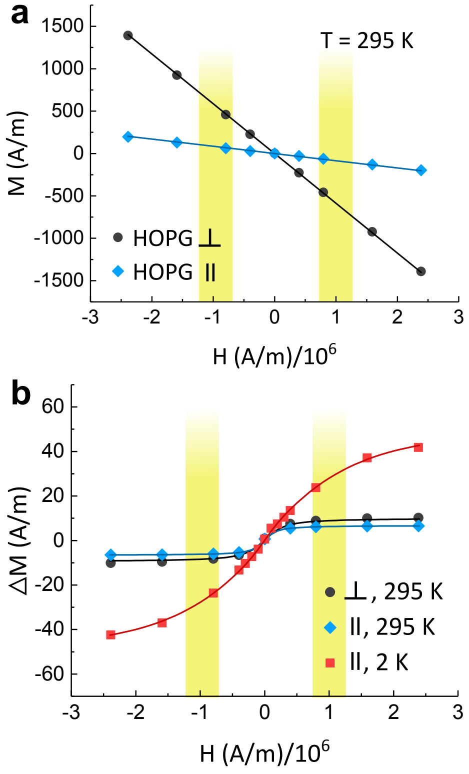

FIG. S1. SQUID measurements of magnetisation M versus magnetic field H on bulk HOPG. **a** Measurements at 295 K for an applied field parallel (‖, blue diamonds) and perpendicular (⊥, black dots) to the HOPG carbon planes. Solid lines: fits considering the sum of a diamagnetic and a ferro-/paramagnetic contribution. **b** Para- and ferromagnetic components for in-plane (blue diamonds) and out-of-plane (black dots) directions at 295 K, and in-plane (red squares) at 2 K, obtained by subtracting the main diamagnetic contribution from data in A). Solid lines: fits to a single (295 K) and double (2 K) Langevin function plus a constant. Yellow areas: range of magnetic field amplitudes used in magnetic transport experiments.

Although the linear diamagnetic response is clearly dominant, after subtraction of the main diamagnetic component, the SQUID data reveal an additional magnetic contribution (possibly ferromagnetic) in both the

www.pnas.org/cgi/doi/10.1073/pnas.1817989116

HOPG type	$\chi_\perp/10^{-4}$	$\chi_\parallel/10^{-5}$	Reference
bulk	-5.82 ± 0.01	-8.2 ± 0.1	this work
bulk	-4.5	-8.5	[1]
bulk	-8.2	-1.1	[2]

TABLE S1. HOPG dimensionless volume magnetic susceptibility (SI units) measured here and reported in the literature.

in-plane and out-of-plane directions (Figure S1b, black and blue points). Measurements at 2 K for the in-plane component (red squares in Figure S1b) show the presence of a larger contribution that corresponds to the sum of: a weak contribution that saturates at low fields ($< 5\times10^5$ A/m) and is similar in magnitude and shape to the one observed at 295 K (possibly ferromagnetic), and an additional larger paramagnetic contribution not apparent at 295 K due to the inverse relation between paramagnetic magnetic susceptibility and temperature [3]. The data hence point towards the presence of a small paramagnetic contribution arising from defects and/or vacancies in the material (see below) and apparent only at low temperature, together with a temperature-independent small ferromagnetic contribution likely due to impurities, similar to that described in ref. [4] (see below). These contributions, however, can be neglected in comparison to the overall diamagnetic properties of bulk HOPG in the range of temperatures and external fields (yellow areas in Figure S1) used in this study.

Weak ferromagnetic contributions have been previously reported for pristine bulk HOPG [5, 6]. Specifically, local ferromagnetism was measured near line defects and grain boundaries using magnetic force microscopy [6]. However, a later study [4] observed no intrinsic defect-induced ferromagnetism in pristine HOPG samples from various manufacturers and grades characterised with SQUID and backscattering scanning electron microscopy (SEM). They found that the weak ferromagnetism ($\Delta M < 3 \times 10^{-3}$ emu/g) observed at room temperature was due to the presence of micron-sized magnetic impurities (oxidised Fe and Ti) buried under the sample surface with typical in-plane separations of 100-200 μm [4]. The authors point out that these impurities can be easily missed if SEM is not used, as might be the case for previous studies [5, 6].

Regarding the weak paramagnetic contribution measured, it has been reported that vacancies generated by ion bombardment in graphene carry spin-1/2 magnetic moment and can lead to paramagnetism [4]. Sharp electronic resonances (associated with localised unpaired electrons and local magnetic moments) have been measured around isolated irradiation vacancies in HOPG using atomic-resolution scanning tunneling microscopy [7]. The fact that vacancy defects can result in a net magnetic moment has also been demonstrated theoretically [8, 9].

As measuring the magnetisation of a single HOPG microflake is beyond the sensitivity of SQUID, it is not easy to elucidate whether the diamagnetism of an individual microflake is exactly the same as that of bulk HOPG or if there is any significant perturbation owing to the sonication step in the particle preparation process. As HOPG diamagnetism relies on mobile, delocalised π electrons in the carbon lattice, the possible presence of localised defects and the possibly smaller (nano) crystallite sizes in sonicated microflakes could in principle lead to disturbed electron delocalisation and reduced diamagnetism. However, given the Raman spectroscopy evidence (see below), this is unlikely.

SUPPLEMENTARY NOTE 2: SIZE OF HOPG MICROFLAKES

The microflakes are approximated as elliptical discs with half-sizes a, b and c (see Fig. S4 below). The dimensions of the elliptical surface semi-axes (a, b) are obtained by processing the acquired microscopy images of particle transport (Fig. S2) with single-particle-tracking algorithms (main text, Methods). a and b are obtained as the mean ellipse half-sizes from values detected for all suitable frames, for all trajectories corresponding to a given particle. The particle half-thickness c is estimated to be $\sim$0.6 μm from previous measurements with similar HOPG micro-flakes extracted with a higher-magnification (40$\times$) objective [10]. Dimensions and calculated volumes ($2\pi abc$) for all HOPG microflakes used in experiments are shown in Table S2, with the average dimensions (full sizes) being $\sim$4.6 μm $\times$ 3.4 μm $\times$ 1.2 μm. The relative uncertainty in particle volume is 25-27%.

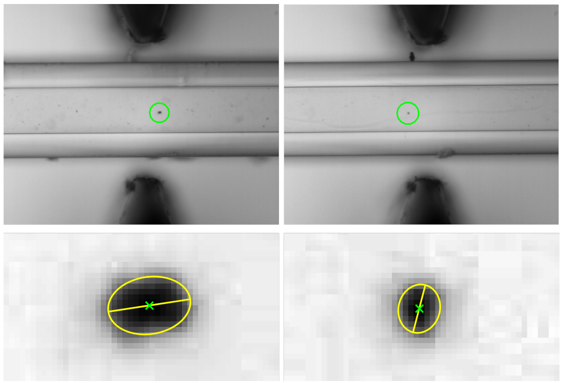

FIG. S2. Microscope images of HOPG microflakes acquired with a 10$\times$ objective. The top row shows full frames with the tracked microflake inside a green circle for particles B1 (left) and B2 (right) (Table S2). The bottom row shows a corresponding zoom into the tracked particle, with centre of mass marked as a green cross and best-fit ellipse in yellow.

sample	particle	$a(\mu\text{m})$	$b(\mu\text{m})$	$c(\mu\text{m})$	$V(\mu\text{m}^3)$	k
B	B1	3.8±0.1	2.2±0.1	0.60±0.15	32±8	a
	B2	1.9±0.1	1.4±0.1	0.60±0.15	10±3	b
	B3	1.5±0.1	1.1±0.1	0.60±0.15	6±2	$a+b$
C	C1	2.9±0.1	2.2±0.1	0.60±0.15	24±6	$a+b$
	C2	2.5±0.1	2.0±0.1	0.60±0.15	19±5	$a+b$
	C3	1.8±0.1	1.5±0.1	0.60±0.15	10±3	$a+b$
	C4	2.4±0.1	1.9±0.1	0.60±0.15	17±4	b
	C5	1.8±0.1	1.3±0.1	0.60±0.15	8±2	b

TABLE S2. Particle sizes (elliptical disc semi-axes a, b and c) for all HOPG microflakes used in experiments (samples in Table I, main text). Uncertainties are standard deviations for data extracted from all frames in all videos corresponding to each particle. The indicated k is the direction of motion for each particle in experiments.

SUPPLEMENTARY NOTE 3: DETAILS OF RAMAN SPECTROSCOPY OF HOPG

Figure S3 compares averaged Raman spectra for the HOPG bulk (average of 36 spectra acquired over a grid area $10\,\mu$m $\times$ $10\,\mu$m with $2\,\mu$m spacings) and for an individual representative HOPG microflake produced by 1 hour of sonication (average of 4 spectra acquired with $2\,\mu$m grid spacing for a microflake with approximate lateral dimensions $8\,\mu$m $\times$ $5\,\mu$m).

The Raman spectrum for graphite and HOPG has been extensively studied [11–14]. In pristine, high-grade bulk HOPG or single-crystal graphite, in absence of a significant number of structural defects, the main peaks in the spectrum are the G peak at $\sim$1580 cm^{-1} and the 2D peak (second order of the D peak) at $\sim$2700 cm^{-1}, as shown for our bulk HOPG in Fig. S3a. The spectra for the HOPG microflake in Fig. S3b show an increased level of disorder compared to the bulk, evidenced by the appearance of a small D peak at $\sim$1350 cm^{-1}. Disorder can take the form of point defects in the carbon lattice and/or increased edges and borders, e.g., finite-size effects owing to smaller in-plane crystallite domain sizes [11–13], surface roughness, etc. On visual inspection under the microscope, surface roughness is larger for the sonicated microflake compared to the bulk. The sample edges always give rise to a non-zero D peak (even for intact structures) [12]. Care was taken for the laser spot to avoid the sample edges and visually smoothest surfaces were chosen to collect the spectra.

The ratio of the D peak intensity to that of the G peak, $I_\mathrm{D}/I_\mathrm{G}$, is an indication of the amount of disorder in the sample. Values of $I_\mathrm{D}/I_\mathrm{G}$ ratios for all measured spectra are calculated for both the HOPG bulk and microflake samples. $I_\mathrm{D}/I_\mathrm{G}$ values are close to zero (0.008-0.013) at all positions for bulk HOPG while moderately larger values in the range 0.06-0.11 are measured for the microflake.

The spectra for the microflake are consistent with a transition to nanocrystalline graphite (reduced crystallite sizes compared to bulk HOPG). The absence of a shift in the G peak position and of significant peak broadening indicates that a transition to highly disordered amorphous carbon has not taken place [12]. The fact that a doublet can be just about resolved within the second-order 2D peak indicates that out-of-plane carbon stacking order (ABAB) has not been lost [15]. Our measurements therefore indicate that the microparticle preparation process (notably the bath sonication step) induces a moderate disorder in the HOPG structure but most probably does not modify the diamagnetic properties of the microflakes compared to the bulk.

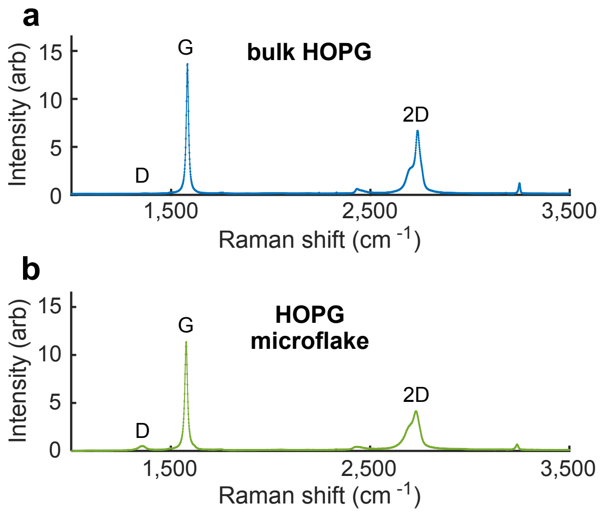

FIG. S3. Raman spectroscopy of HOPG. **a)** Averaged spectrum measured for HOPG bulk. **b)** Averaged spectrum for an individual HOPG microflake produced via 1 hour of sonication.

SUPPLEMENTARY NOTE 4: DETAILED THEORY DERIVATION: MAGNETIC MANIPULATION OF ANISOTROPIC DIAMAGNETIC MICROPARTICLES

We define a particle frame of reference with axes (x, y, z) fixed to the particle so that the xy plane corresponds to the graphene planes and z is normal to these planes. We define also a fixed laboratory frame of reference with axes (X, Y, Z), as shown in Fig. S4.

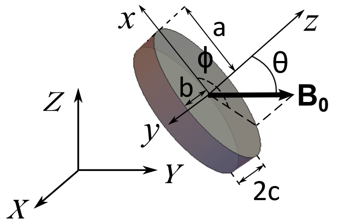

FIG. S4. Schematic of HOPG microflake (approximated as an elliptical disc) in the presence of a magnetic field B_0.

In the particle frame of reference, the volume magnetic

susceptibility tensors for the fluid ($\boldsymbol{\chi}_1$) and particle ($\boldsymbol{\chi}_2$) are expressed as:

$$\boldsymbol{\chi}_1 = \begin{pmatrix} \chi_1 & 0 & 0 \\ 0 & \chi_1 & 0 \\ 0 & 0 & \chi_1 \end{pmatrix}, \; \boldsymbol{\chi}_2 = \begin{pmatrix} \chi_{2,x} & 0 & 0 \\ 0 & \chi_{2,y} & 0 \\ 0 & 0 & \chi_{2,z} \end{pmatrix}, \quad (1)$$

where χ_1 is the isotropic magnetic susceptibility of the fluid and $\chi_{2,x}$, $\chi_{2,y}$ and $\chi_{2,z}$ are the components of the anisotropic magnetic susceptibility of the particle. For HOPG, we have $\chi_{2,x} = \chi_{2,y} \equiv \chi_{2,\parallel}$ (in-plane) and $\chi_{2,z} \equiv \chi_{2,\perp}$ (out-of-plane).

The applied static magnetic field $\mathbf{B}_0$ makes an angle θ to the out-of-plane z axis and its projection onto the xy plane makes an angle ϕ to the x axis (Fig. S4). Hence, we can write: $\mathbf{B}_0 = B_0(\sin\theta\cos\phi\,\hat{\mathbf{x}} + \sin\theta\sin\phi\,\hat{\mathbf{y}} + \cos\theta\,\hat{\mathbf{z}})$, where B_0 is the field amplitude. The magnetic field is assumed to be uniform over the particle size. In the presence of $\mathbf{B}_0$, the submerged particle acquires an effective induced magnetic moment given by [16]:

$$\mathbf{m}_{\text{eff}} = \frac{V}{\mu_0}(\boldsymbol{\chi}_2 - \boldsymbol{\chi}_1)\cdot\mathbf{B}_{\text{in}}\,, \quad (2)$$

where V is the particle volume, μ_0 is the permeability of free space and $\mathbf{B}_{\text{in}}$ is the magnetic field inside the particle, with components $B_{\text{in},k}$ ($k = x, y, z$) given by:

$$B_{\text{in},k} = \frac{\mu_1}{\mu_1 + (\mu_{2,k} - \mu_1)L_k} B_{0,k}\,. \quad (3)$$

Here, $\mu_{2,k} = 1 + \chi_{2,k}$ and $\mu_1 = 1 + \chi_1$ are the relative magnetic permeabilities of particle and fluid, respectively, $B_{0,k}$ are the components of the applied magnetic field and L_k ($0 \leq L_k \leq 1$) are the so-called demagnetising factors that account for particle geometry and magnetisation (these are equivalent to the so-called depolarisation factors in electric field problems [16, 17]). In the particular case of diamagnetic materials ($\chi < 0$), the volume magnetic susceptibilities are very small ($|\chi| < 10^{-4}$), and therefore we have that, in the denominator, $(\mu_{2,k} - \mu_1)L_k = (\chi_{2,k} - \chi_1)L_k \ll \mu_1$. This means that the demagnetising field inside the particle is negligibly small and we can approximate $B_{\text{in},k} \approx B_{0,k}$. As a consequence, the effective magnetic moment induced on the diamagnetic particle is essentially independent of particle shape and geometry and hence can be simplified to:

$$\mathbf{m}_{\text{eff}} \approx \frac{V}{\mu_0}(\boldsymbol{\chi}_2 - \boldsymbol{\chi}_1)\cdot\mathbf{B}_0\,. \quad (4)$$

The magnetic potential energy can be expressed as:

$$U_{\text{m}} = -\int_0^{B_0} \mathbf{m}_{\text{eff}}(B)\cdot \mathrm{d}\mathbf{B} = -\frac{VB_0^2}{2\mu_0}\left[(\chi_{2,\parallel} - \chi_1) + (\chi_{2,\perp} - \chi_{2,\parallel})\cos^2\theta\right], \quad (5)$$

and the magnetic force derived from the potential is:

$$\mathbf{F}_{\text{m}} = -\nabla U_{\text{m}} = \frac{V}{\mu_0} B_0 \nabla B_0 \left[(\chi_{2,\parallel} - \chi_1) + (\chi_{2,\perp} - \chi_{2,\parallel})\cos^2\theta\right] \quad (6)$$

SUPPLEMENTARY NOTE 5: MAGNET CHARACTERISATION AND MAGNETIC FIELD FOCUSING

Figure S5a shows the magnetic field strength for a single block magnet (NdFeB permanent magnet, grade N50M, $25\times25\times20$ mm block, part no. NIBL01484 from MagnetSales) measured with a Gaussmeter probe as a function of distance away from the magnet pole face. The field and field gradient near the magnet pole are, respectively, ~0.5 T and ~50 T/m. Figure S5b shows the measured magnetic field strength for a single magnet block with a focusing iron wedge attached to its surface. The magnetic field near the wedge tip is > 0.7 T and the field gradient is more pronounced (> 500 T/m), thanks to the focusing wedge.

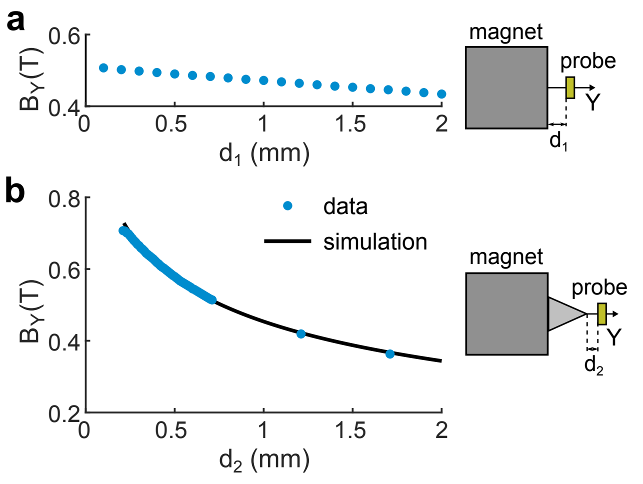

FIG. S5. Characterisation of permanent magnets. **a** Magnetic field strength of a single NdFeB magnet measured with a transverse Hall probe vs distance to the magnet pole surface. **b** Magnetic field strength near a magnet with attached focusing steel wedge vs distance to the wedge tip. Data points: measurements with Hall probe. Solid line: simulated values.

SUPPLEMENTARY NOTE 6: PHOTOGRAPH OF EXPERIMENTAL SET-UP

Figure S6 shows a photograph of the experimental set-up used for magnetophoresis experiments.

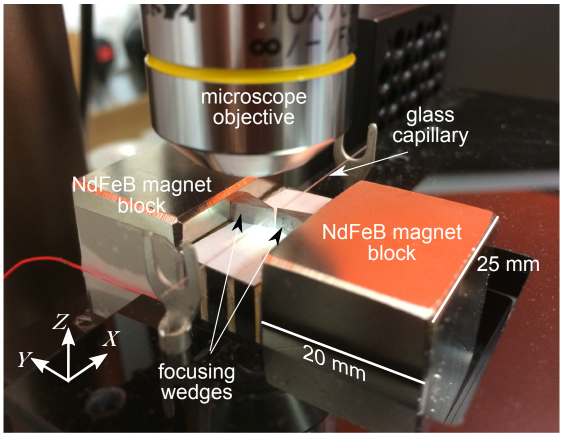

FIG. S6. Set-up for magnetic transport of diamagnetic microparticles in solution. Two NdFeB magnet blocks (opposite poles facing each other) and focusing steel wedges generate a strong magnetic field and field gradient. Acrylic-glass spacers keep the block magnets apart. Submerged microflakes inside a thin glass capillary located in the gap between the wedges move away from the field maximum along X. Capillary and magnets are on independent 3D translation stages for precise positioning. Particle motion is recorded with a microscope objective and camera.

[1] M. D. Simon, L. O. Heflinger, and A. K. Geim, "Diamagnetically stabilized magnet levitation," Am. J. Phys. **69**, 702 (2001).

[2] M. Sepioni, R. R. Nair, S. Rablen, J. Narayanan, F. Tuna, R. Winpenny, A. K. Geim, and I. V. Grigorieva, "Limits on Intrinsic Magnetism in Graphene," Phys. Rev. Lett. **105** (2010).

[3] M. Sepioni, R. R. Nair, S. Rablen, J. Narayanan, R. W. F. Tuna, A. K. Geim, and I. V. Grigorieva, "Limits on Intrinsic Magnetism in Graphene," Phys. Rev. Lett. **105**, 207205 (2010).

[4] R. R. Nair, M. Sepioni, I.-L. Tsai, O. Lehtinen, J. Keinonen, A. V. Krasheninnikov, T. Thomson, A. K. Geim, and I. V. Grigorieva, "Spin-half paramagnetism in graphene induced by point defects," Nature Physics **8**, 199 (2012).

[5] P. Esquinazi, R. H. A. Setzer, C. Semmelhack, Y. Kopelevich, D. Spemann, T. Butz, B. Kohlstrunk, and M. Losche, "Ferromagnetism in oriented graphite samples," Phys. Rev. B **66**, 024429 (2002).

[6] J. Cervenka, M. I. Katsnelson, and C. F. J. Flipse, "Room-temperature ferromagnetism in graphite driven by two-dimensional networks of point defects," Nature Physics **5**, 840 (2009).

[7] M. M. Ugeda, I. Brihuega, F. Guinea, and J. M. Gomez-Rodriguez, "Missing Atom as a Source of Carbon Magnetism," Phys. Rev. Lett. **104**, 096804 (2010).

[8] O. V. Yazyev, "Magnetism in disordered graphene and irradiated graphite," Phys. Rev. Lett. **101**, 37203 (2008).

[9] R. Faccio, H. Pardo, P. A. Denis, R. Y. Oeiras, F. M. Arajo-Moreira, M. Verssimo-Alves, and A. W. Mombr, "Magnetism induced by single carbon vacancies in a three-dimensional graphitic network," Phys. Rev. B **77**, 035416 (2008).

[10] J. Nguyen and Jonathan G. Underwood and I. Llorente Garca, "Orienting lipid-coated graphitic micro-particles in solution using AC electric fields: A new theoretical dual-ellipsoid Laplace model for electro-orientation," Colloids and Surfaces A: Physicochemical and Engineering Aspects **549**, 237–251 (2018).

[11] F. Tuinstra and J. L. Koenig, "Raman Spectrum of Graphite," J. Chem. Phys. **53**, 1126 (1970).

[12] A. C. Ferrari, "Raman spectroscopy of graphene and graphite: Disorder, electronphonon coupling, doping and nonadiabatic effects," Solid State Communications **143**, 47–57 (2007).

[13] L. G. Canado, A. Jorio, E. H. M. Ferreira, F. Stavale, C. A. Achete, R. B. Capaz, M. V. O. Moutinho, A. Lombard, T. S. Kulmala, and A. C. Ferrari, "Quantifying Defects in Graphene via Raman Spectroscopy at Different Excitation Energies," Nano Lett. **11**, 31903196 (2011).

[14] P. H. Tan, Y. M. Deng, and Q. Zhao, "Temperature-dependent Raman spectra and anomalous Raman phenomenon of highly oriented pyrolytic graphite," Phys. Rev. B **58**, 5435 (1998).

[15] H. Wilhelm, M. Lelaurain, E. McRae, and B. Humbert, "Raman spectroscopic studies on well-defined carbonaceous materials of strong two-dimensional character," J. Appl. Phys **84**, 6552 (1998).

[16] T. B. Jones, *Electromechanics of Particles* (Cambridge University Press, 1995).

[17] B. I. Bleaney and B. Bleaney, *Electricity and Magnetism* (Oxford University Press, 1965).
